# Irrelevance of Microsatellite Instability in the Epidemiology of Sporadic Pancreatic Ductal Adenocarcinoma

**DOI:** 10.1371/journal.pone.0046002

**Published:** 2012-09-21

**Authors:** Luigi Laghi, Stefania Beghelli, Antonino Spinelli, Paolo Bianchi, Gianluca Basso, Giuseppe Di Caro, Anna Brecht, Giuseppe Celesti, Giona Turri, Samantha Bersani, Guido Schumacher, Christoph Röcken, Ilona Gräntzdörffer, Massimo Roncalli, Alessandro Zerbi, Peter Neuhaus, Claudio Bassi, Marco Montorsi, Aldo Scarpa, Alberto Malesci

**Affiliations:** 1 Laboratory of Molecular Gastroenterology, Humanitas Clinical and Research Center, Rozzano, Milan, Italy; 2 Department of Gastroenterology, Humanitas Clinical and Research Center, Rozzano, Milan, Italy; 3 ARC-NET, Centre for Applied Research on Cancer, University of Verona, Verona, Italy; 4 General Surgery, IRCCS Istituto Clinico Humanitas, Rozzano, Milano, Italy; 5 School of Molecular Medicine, University of Milan, Milan, Italy; 6 School of Experimental Pathology and Neuropathology, University of Milan, Milan, Italy; 7 Department of General Surgery and Transplantation, Charitè Campus Virchow, University of Berlin, Berlin, Germany; 8 Department of Pathology and Diagnostics, University of Verona, Verona, Italy; 9 Department of Pathology, Charitè Campus Virchow, University of Berlin, Berlin, Germany; 10 Department of Pathology, Humanitas Clinical and Research Center, Rozzano, Milan, Italy; 11 Department of Medical Biotechnology and Translational Medicine, University of Milan, Milan, Italy; 12 Department of Surgery and Oncology, University Hospital Trust of Verona, Verona, Italy; University of Bari & Consorzio Mario Negri Sud, Italy

## Abstract

**Background and Aims:**

Pancreatic cancer risk is increased in Lynch syndrome (LS) patients with mismatch repair gene defects predisposing to colonic and extracolonic cancers with microsatellite instability (MSI). However, the frequency of MSI pancreatic cancers has never been ascertained in consecutive, unselected clinical series, and their contribution to the sporadic and inherited burden of pancreatic cancer remains to be established. Aims of the study were to determine the prevalence of MSI in surgically resected pancreatic cancers in a multicentric, retrospective study, and to assess the occurrence of pancreatic cancer in LS.

**Methods:**

MS-status was screened by a panel of 5 mononucleotide repeats (*Bat26, Bat25, NR-21, NR-24* and *NR-27*) in 338 consecutive pancreatic ductal adenocarcinoma (PDAC), resected at two Italian and one German referral centres. The personal history of pancreatic cancer was assessed in an independent set of 58 probands with LS and in 138 first degree relatives who had cancers.

**Results:**

Only one PDAC (0.3%) showed MSI. This was a medullary type cancer, with hMLH1-deficiency, and no identified germ-line mutation but methylation of *hMLH1*. Pancreatic cancer occurred in 5 (2.5%) LS patients. Histological sampling was available for 2 cases, revealing PDAC in one case and an ampullary cancer in the other one.

**Conclusions:**

MSI prevalence is negligible in sporadic, resected PDAC. Differently, the prevalence of pancreatic cancer is 2.5% in LS patients, and cancers other than PDAC may be encountered in this setting. Surveillance for pancreatic cancer should be advised in LS mutation carriers at referral centers.

## Introduction

Despite ranking fourth for incidence, pancreatic ductal adenocarcinoma (PDAC) has the first mortality rate among gastrointestinal cancers [1]. Knowledge of the molecular basis of PDAC, including genetically determined predispositions, increased steadily in the last decade. [2]. The syndromes predisposing to PDAC have a tumor spectrum not restricted to the pancreas, and comprise melanoma in carriers of *p16/CDNK2* germline mutations, and breast cancer in carriers of *BRCA1* or of *BRCA2* mutations. The risk of developing PDAC also increases in inherited predispositions to colorectal cancer, namely the Peutz-Jegers and the Lynch syndrome (LS, or Hereditary Non-Polyposis Hereditary Colorectal Cancer) [2], [3]. LS is caused by germ-line mutations in one of the mismatch repair (MMR) genes *hMLH1, hMSH2,* and, less frequently, *hMSH6* or *PMS2*. Cancers with MMR defects, whether arising in LS patients or sporadically, due to somatic hMLH1 hypermethylation, typically show the molecular phenotype of microsatellite instability (MSI) [4]. MSI prevalence approaches 10% in colorectal cancer, LS accounting for one third of the cases and *hMLH1* hypermethylation for the remaining part [4], [5]. Typically, MSI cancers beside exhibiting peculiar pathological features such as medullary histology [6], have a lower pathological stage at diagnosis, and thus a better prognosis [5]. MSI prevalence in gastric, uterine and ovarian cancers approaches that of colon cancer [7], [8]. Considering the ominous prognosis of pancreatic cancer [9], [10], it would be relevant whether MSI testing could identify PDAC patients with better survival [11], [12]. However, the prevalence of MSI remains undefined in pancreatic cancer.

Studies based upon the review of family history, found a risk of pancreatic cancer 7–8 times higher in LS families than in the general population [13], [14]. On the other hand, a few studies assessed the prevalence of MSI in PDAC specimens. Goggins first reported 3 (3.7%) MSI cases in a North American series comprising 82 PDAC [15], while European studies from Poland [16] and Italy [17] did not find any MSI or MMR deficient PDAC in small surgical series. Studies of pre-selected cases detected MSI in 3 (8.6%) out of 35 PDAC from long-term (>3 years) survivors [18], and in 4 (22.0%) out of 18 PDAC with medullary histology [19]. Rather differently, studies in unselected Japanese PDAC reported MSI rates above 10% [11], [12] (Table 1). Notably, the MSI phenotype has been mainly found in *K-RAS* wild-type PDAC [12], [15].

**Table 1:** Published studies on the frequency of the MSI or MMR-defective phenotype in Pancreatic Ductal Adenocarcinoma.

						MS-Status Assessment					
		Patients				Microsatellite markers		MMR deficiency			
Author(year)	Ref.		Series	MSI cancers		Mono- nucleotides	Di-nucleotides	hMLH1	hMSH2	LynchSyndrome	Country
		n		n	(%)	n	n	n	n	n	
Goggins(1998)	[15]	82	Unspecified*	3	3.7	1	4	-	-	0	U.S.
Ghimenti(1999)	[17]	21	Unspecified	0	0	0	10	0#	0#	0	Italy
Wilentz(2000)	[19]	18	Selected(Medullary )§	4	22.0	2	1	4	0	0″	U.S.
Yamamoto(2001)	[12]	103	Partiallyselected°	16°	15.5	2	3	8	0#	3	Japan
Nakata(2002)	[11]	46	Unspecified	8	17.4	0	8	-	-	-	Japan
Tomaszewska (2003)	[16]	30	Unspecified	-	-	-	-	0	0	0	Poland
Maple(2005)	[18]	35	Selected (Long survivors)∧	3	8.6	4	4	2	1	2	U.S.

* Tumor specimens passed through xeno-transplantation of PDAC, unspecified whether consecutively collected.

# By genomic DNA analysis for mutations.

§ Medullary cancers selected out of 450 randomly chosen PDAC.

“One patient with positive Bethesda criteria but negative *hMLH1* mutational analysis.

°3 PDAC arising in LS patients added to a series of 100 patients, unspecified whether consecutive.

∧ Long survivors (≥3 years) selected out of 373 PDAC patients.

Currently, the negative studies on MSI or MMR defects in PDAC [16], [17] appear underpowered to detect significant difference (power<0.60 with 0.05 α-level *vs*. ref. 15). Thus, the relevance of MSI in PDAC remains questionable, due to the discrepancies among the studies and to the limited data available in an evidence-based perspective [20]. Accordingly, the aim of our study was to assess the MSI prevalence in a large, multicentric and consecutive series of western European patients with pancreatic cancer, well characterized for pathological and molecular features, and for patient survival. In parallel, we also assessed the frequency of pancreatic tumors in a series of LS probands and of their first degree relatives retrieved from a colorectal cancer family clinic.

## Results

### Prevalence and Clinico-pathological Features of MSI PDAC

**Figure 1:**
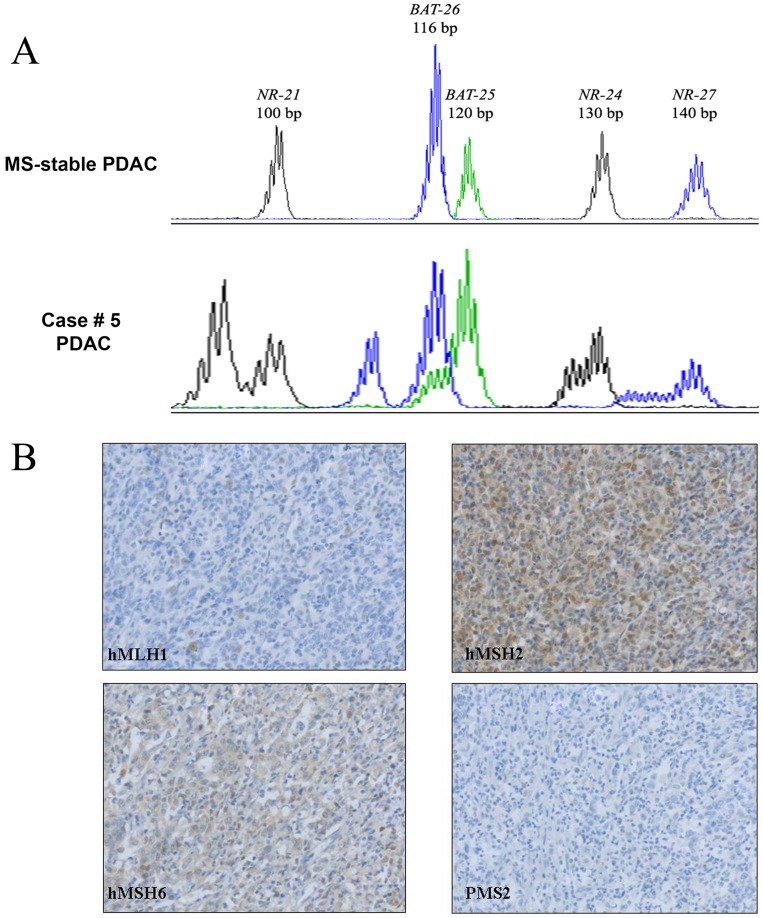
Electropherograms of the analysis of mononucleotide microsatellite markers *BAT26, BAT25, NR-21, Nr-24* and *NR-27* in pancreatic cancers. From top to bottom, an example of MS-Stable PDAC, and the only MSI PDAC (Case # 5) (Panel A). Immunohistochemical analysis of MMR protein expression in the medullary hMLH1-deficient PDAC (Case #5), retaining hMSH2 and hMSH6 expression. Note the loss of PMS2, due to protein degradation in the absence of the cognate partner hMLH1 (Objective magnification, 10×) (Panel B).

Only 1 (0.3%) cancer out of 338 PDAC showed MSI in all the tested quasimonomorphic mononucleotide repeats (*Bat25, Bat26, NR-21, NR-24* and *NR-27*). The other 337 sample did not show instability at any of the 5 markers (Figure 1, Panel A). The patient with MSI, hMLH1-deficient PDAC, was a 79 years old female, with no history of familiar cancer. The cancer, arisen in the pancreatic head, had a diameter of 4.5 cm, involving the distal pancreatic duct and the duodenum. This pT4N1b hMLH1-deficient cancer was poorly differentiated (Figure 1, Panel B), and angioinvasive. The patient died of post-surgical complications. No *hMLH1* mutations were detected in DNA extracted from normal tissue retrieved from pathological specimens, but *hMLH1* promoter showed hypermethylation. Cancer tissue harbored mutated *K-RAS^cod12^* and wild-type *B-RAF*. Furthermore, none of the 157 tested PDAC harbored *B-RAF^V600E^* mutation.

### Prevalence of Pancreatic Tumors in LS

We identified 58 LS probands; 35 (60.3%) with hMSH2 defects, 20 (34.5%) with hMLH1 defects, and 3 (5.2%) with *hMSH6* defects, plus 145 first degree relatives (35 in families with *hMLH1*, 98 in families with *hMSH2*, and 12 in families with *hMSH6* defects) with a personal history of cancer. Out of a total of 203 LS patients, 5 (2.5%) had pancreatic tumors. The 5 tumors occurred in 5 families with *hMSH2* defects, and 4 tumors were diagnosed below the age of 50 years. Tumor sampling was available for 2 cases from mutation carriers. In one case cytology obtained by fine-needle was diagnostic for PDAC, but no tissue was available for MS-status testing. The other case, treated by resection of the pancreatic head, was an ampullary cancer, with histological features of pancreato-biliary poorly-differentiated (G3) adenocarcinoma, showing MSI and hMSH2 deficiency.

## Discussion

MSI was an extremely rare event in the largest and only consecutive series of PDAC ever studied. The implications of our results involve assessment methods, pathological features, and clinical behavior of MSI PDAC as reported so far.

A debated issue has been the technical reproducibility and accuracy of the methods to test MS-status [4]. Already the first proposed microsatellite panel was aimed to standardize MS-status assessment [21], although the di-nucleotide markers initially employed generate false positives [4], [22], while mononucleotides are specific [4], [23] and can detect MSI [24] without matched normal tissue [4], [22]. Noteworthy, the 5 employed markers were fully concordant in all MS-stable (or -unstable) cases. Accordingly, testing multiple mononucleotide repeats does not increase sensitivity and specificity in a cancer type in which MSI phenotype is so rarely encountered. At any event, our study is the only one performed by using a standard panel of 5 mononucleotide repeats [4], [23], [25] on the largest series ever investigated. Accordingly, our method reflects the true prevalence of MSI cancers. MSI prevalence 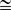
20% such as reported by Nakata et al. using di-nucleotide markers only and not supported by immuno-histochemistry is questionable [11]. However, MS-status assessment by mono-nucleotide markers detected a striking 13% prevalence of MSI PDAC, classified as sporadic, in Japanese patients [12]. Thus, our data definitely establish that the prevalence of MSI PDAC in western countries does not exceed 1%, [15]–[18], while further studies are needed to confirm in consecutive series a high prevalence of MSI PDAC in Japanese.

PDAC accounts for the lowest 5-year survival of any cancer. Although surgery is considered to offer the only chance of cure, the mean survival after resection is merely 18–20 months, and the 5-years survival rate does not exceed 20% (for stage I/II) in Western world [1], [9], [26]. Differently, the survival rate in Japan is close to 40% [27]–[30], and studies conducted in US revealed a longer survival for Asian patients with PDAC [31]. A higher proportion of less advanced cancers in Asian than in Western patients [32], to which MSI cases might contribute, would explain this difference. Even though this might be the case in Japanese [12], in Western countries less then 10% of PDAC long survivors had MMR-deficient cancers [18], and our data rule out the possibility that MSI cases substantially contribute to improved survival. Finally, we cannot rule out a different MSI prevalence in advanced PDAC not amenable to surgical resection, currently the vast majority. However, due to the extremely poor survival of patients with advanced PDAC at diagnosis [1], the prevalence of MSI cases should be lower in these patients than in those undergoing surgery.

The only MSI and hMLH1-deficient PDAC which we detected showed *hMLH1* promoter hypermethylation and was the only medullary cancer in our series. Following the description by Goggins and coll. of pancreatic medullary histology as a feature of MSI PDAC [15], others reported medullary MSI PDAC in patients with a family history consistent with LS [19], [33]. Our case finding confirms that the medullary phenotype is a feature of MSI PDAC, irrespectively of inherited or sporadic origin [19], which occurs without *BRAF^V600E^* mutation in the latter [34]. The lack of *BRAF^V600E^* mutation in our series also rules out any alternative role to *K*-*RAS* for this oncogene in pancreatic carcinogenesis, irrespectively of tumor MS-status [35].

**Table 2:** Patient demographics and survival, family history of gastrointestinal cancer, tumor pathological and molecular features, in 338 consecutively resected PDAC.

Features		PDAC(n = 338)
		n (%)
**Patient Age** (mean ± S.D.)		62.5±9.9
**Patient Gender**	Male	161 (47.6%)
	Female	177 (52.4%)
**Patient survival**	>3 years	42 (12.4%)
	≤3 years	296 (87.6%)
**Family History** **of GI Cancer** *	Yes	48 (14.2%)
	No	290 (85.8%)
**Tumor Location**	Head	266 (78.7%)
	Body	37 (10.9%)
	Tail	35 (10.4%)
**Tumor Staging**	I	15 (4.4%)
	II	72 (21.3%)
	III	220 (65.1%)
	IVa	20 (5.9%)
	IVb	11 (3.3%)
**Tumor Grading**	G1	15 (4.4%)
	G2	187 (55.3%)
	G3	126 (37.3%)
	G4°	1 (0.3%)
	Mucinous	9 (2.7%)
	NA	0
**Medullary Phenotype**	Yes°	1 (0.3%)
	No	337 (99.7%)
**K-RAS** ***^cod.12^***	Mutated	287 (84.9%)
	Wild-Type	51 (15.1%)

* Including stomach, colon and pancreatic cancer in first degree relatives.

°Reported as G4 with respect to Tumor Grade.

As opposed to the rarity of sporadic MSI PDAC, pancreatic cancer would occur more frequently in in LS patients than in the general population. The 2.5% frequency of pancreatic tumors in our LS series was almost identical to the 3.0% of unspecified pancreatic cancer reported by Geary, a 7-fold higher occurrence than expected [13]. Kastrinos calculated a 8.6 fold increased risk and 3.7% life-time risk of pancreatic cancer in LS families based upon family reports [14]. Accordingly, germ-line MMR defects predispose to pancreatic cancer. One tumor in the Italian series of LS was a poorly differentiated pancreato-biliary subtype ampullary cancer, tipically associated with MSI [36]. This occasional finding rises the hypothesis that tumors other than PDAC might contribute to the high rate of not histologically confirmed pancreatic cancer in LS. At any event, pancreatic surveillance for neoplasms in carriers of MMR gene mutation should be advised in referral centers, especially for LS families with a positive history of pancreatic tumors [13], [14]. A similar approach aimed to increase the rate of early diagnosis, and thus of surgical management, will also provide insights on the survival gain due to the proper recognition and treatment of pancreatic cancer in the setting of LS.

## Materials and Methods

### Tumor Series

For the retrospective study, a total of 338 consecutive specimens from Europeans patients who had undergone pancreatic resection for PDAC at three academic institutions were retrieved. The series included 181 PDAC from the University Hospital of Verona, 91 from the Humanitas Clinical and Research Center, Rozzano, University of Milan, and 66 from the Charitè Campus Virchow, University of Berlin. Patient demographics and survival, family history of gastrointestinal cancer, and tumor pathological and molecular features of the PDAC series are detailed in Table 2.

**Table 3:** Sequences of the primers employed for *BRAF^V600E^* analysis.

Gene/Exon	Forward Primer	Reverse Primer
*BRAF*	CTACTGTTTTCCTTTACTTACTACACCTCAGA	ATCCAGACAACTGTTCAAACTGATGGGAC
V600E probe	FAM-CTACAGaGAAATCTC	
Wild-type probe	VIC-AGCTACAGtGAAATC	

### Ethics Statement

The written informed consent of patients to the handling of clinical data had been obtained by the referring physicians at admission for surgery. Being the study at no risk to patients and their privacy, the approval for the use of pathology specimens with a waiver of consent was granted by the Review Board of the Humanitas Clinical and Research Center and of the Charitè Campus Virchow, and by the Ethics Committees of the University Hospital Trust of Verona. A coded data-base was prepared by clinical researchers unaware of molecular data, and deidentified samples under code were obtained from the pathology archives for molecular analysis.

### MS-status Assessment and Analysis of MMR Defects

DNA was extracted from 5 micron thick, paraffin-embedded specimens, and cancer tissue was micro-dissected if tumor cells did not account for at least 50% of the sample. MSI assignment was based on the analysis of mononucleotide repeats. After DNA extraction by proteinase-K digestion and phenol-chloroform purification, amplification of the mononucleotide microsatellites *BAT25, BAT26, NR-21, NR-24* and *NR-27* with fluorescent dye-labeled primers was followed by capillary-gel electrophoresis (ABI PRISM 310 DNA Sequencer, Perkin-Elmer, Foster City, CA, USA) [4], [5], [25], [37], [38].

hMLH1 and hMSH2 MMR protein defects were tested by immunohistochemistry in the Verona series, as well as in MSI cases identified by molecular testing. hMLH1 (G-168 monoclonal antibody, PharMingen; San Diego, CA, US), hMSH2 (clone FE 11, Oncogene Sciences; Cambridge, MA, US), hMSH6 (clone 44, Transduction Laboratories), and PMS2 (clone, A16-4, PharMingen) were tested according to previously described methods [5]. Lesions were considered negative in the complete absence of detectable nuclear staining in neoplastic cells. In MSI cases, sequencing of defective MMR genes, according to the protein defect, was performed. Exons and intron/exon boundaries of the defective gene were amplified according to previously described techniques [5].

### *K-RAS^cod12^* Status

Mutations at codon 12 of *KRAS* were detected by PCR–RFLP using a modified primer that creates a restriction site for BstNI (New England Biolabs Inc., Beverly, MA, USA) restriction enzymes [39].

### *BRAF^cod600^* Status

The mutational status of *B-RAF* codon 600 vas determined RT-PCR using a TaqMan SNP Genotyping Assay (Applied Biosystem) in 157 (44.1%) PDAC specimens, representing all consecutive cases collected from the Universities of Milan and Berlin. TaqMan MGB probes were designed using the Custom TaqMan Assay Design Tool (Applied Biosystem). The chosen reporter fluorophores were VIC for detecting the wild type allele and FAM for the mutant allele (Table 3).

### Methylation-specific PCR

Methylation of *hMLH1* CpG islands was determined by treatment with sodium bisulfite and PCR, using specific primers for methylated and unmethylated DNA [40]. PCRs were performed with positive methylation control, a human placental DNA treated *in vitro* with excess of SssI methyltransferase that generates a completely methylated DNA.

### Frequency of Pancreatic Tumors in LS Patients

The occurrence of tumors arising in the pancreas was retrospectively assessed in 58 LS probands with known MMR gene mutations and in 145 first degree relatives for whom a personal history of cancer was available at the Colorectal Cancer Family Clinic of the Humanitas Clinical and Research Center.

The LS series did not overlap with the surgical PDAC series of the same institution.

### Ethics Statement

Lynch syndrome survey had been approved by the Ethycal Committee of the Humanitas Clinical and Research Center. Probands undergone interview and mutation-testing are registered in an institutional review board–approved protocol, and provided written informed consent.
